# Emergent Transcatheter Tricuspid Edge-to-Edge Repair for Right Ventricular Shock From Tricuspid Regurgitation in Hypertrophic Cardiomyopathy

**DOI:** 10.1016/j.jaccas.2025.104448

**Published:** 2025-07-16

**Authors:** Nicholas J. Valle, Amin Yehya, Animesh Rathore, Erich L. Kiehl, Matthew R. Summers

**Affiliations:** a Eastern Virginia Medical School, Macon and Joan Brock Virginia Health Sciences at Old Dominion University, Internal Medicine Residency Program, Norfolk, Virginia, USA; b Sentara Medical Group, Sentara Health, Department of Advanced Heart Failure, Mechanical Circulatory Support, and Transplant Cardiology, Virginia Beach, Virginia, USA; c Eastern Virginia Medical School, Macon and Joan Brock Virginia Health Sciences at Old Dominion University, Department of Cardiology, Norfolk, Virginia, USA; d Sentara Medical Group, Sentara Health, Department of Vascular Surgery, Virginia Beach, Virginia, USA; e Sentara Medical Group, Sentara Health, Department of Electrophysiology, Virginia Beach, Virginia, USA; f Sentara Medical Group, Sentara Health, Department of Structural Cardiology and Complex Coronary Intervention, Virginia Beach, Virginia, USA

**Keywords:** 3-dimensional imaging, cardiomypathy, chronic heart failure, complication, hemodynamics, reduced ejection fraction, tricuspid valve, valve repair

## Abstract

**Background:**

Transcatheter tricuspid edge-to-edge repair (T-TEER) is an emerging technology used to treat severe tricuspid regurgitation (TR). The pivotal trials demonstrating the safety and efficacy of T-TEER exclude patients with severely reduced left ventricular function (≤20%).

**Case Summary:**

We demonstrate the use of T-TEER in a patient with end-stage hypertrophic cardiomyopathy who suffered acute, torrential TR and subsequently developed cardiogenic shock as a complication of high-risk transvenous lead extraction.

**Why Beyond the Guidelines:**

Currently, there are no guidelines addressing the nonsurgical emergent management of acute TR.

**Discussion:**

The TriClip system (Abbott) may have certain advantages when used in managing acute primary TR compared with other valve repair and/or replacement approaches.

**Take-Home Message:**

TriClip can be considered for urgent use to correct severe TR in hemodynamically unstable patients who are not surgical candidates.

## Graphical Abstract

**Figure:**
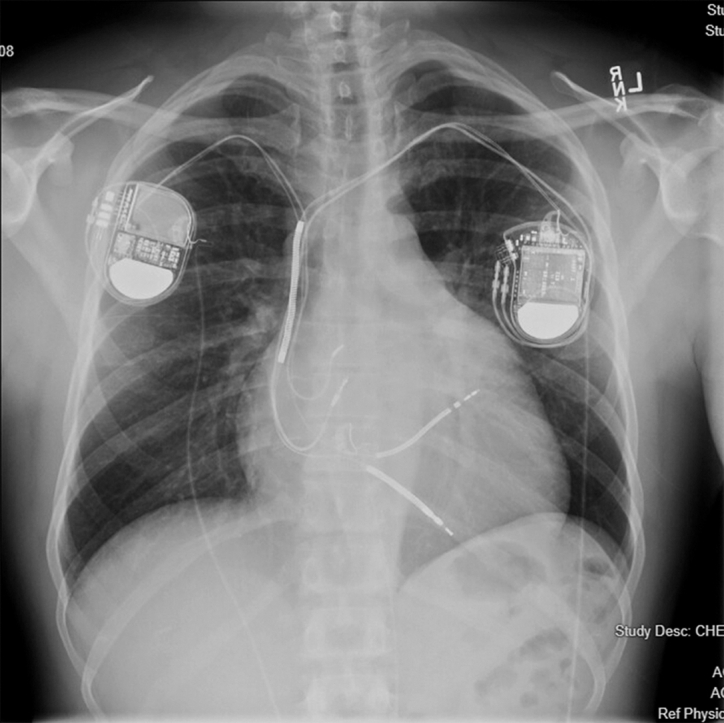

Percutaneous management of tricuspid regurgitation (TR) is a rapidly evolving field.^1^ The TRILUMINATE trial (Trial to Evaluate Cardiovascular Outcomes in Patients Treated with Tricuspid Valve Repair) demonstrated the safety and efficacy of percutaneous transcatheter tricuspid edge-to-edge repair (T-TEER) using the TriClip system (Abbott) for patients with severe TR.^2^ One of the exclusion criteria for this trial included patients with left ventricular ejection fraction (LVEF) ≤20%. Similarly, the Edwards PASCAL Transcatheter Valve Repair System in Tricuspid Regurgitation Early Feasibility Study trial studied the feasibility and safety of T-TEER with the PASCAL system (Edwards), and it excluded patients with LVEF <30%.^3^ The use of T-TEER technology in the management of acute TR in unstable patients with severely reduced LVEF has not been studied. We present the case of a 48-year-old man with end-stage hypertrophic cardiomyopathy (HCM) who developed acute TR and cardiogenic shock as a complication of complex transvenous lead extraction (TLE).

## Case Summary

**Figure 1:**
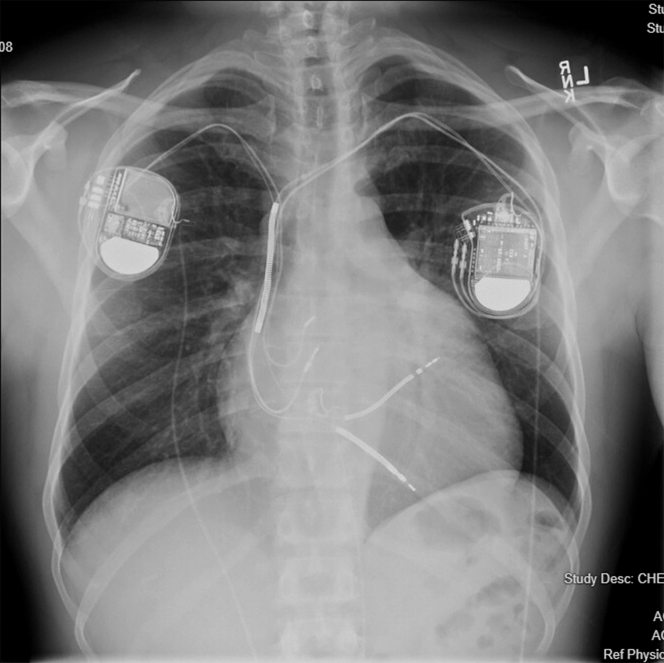
Chest Plain Film Original dual-chamber ICD (patient's left) was placed in 2002, which was complicated by lead fracture, venous occlusion, and abandonment. Replacement dual-chamber ICD (patient's right) was placed in 2017. Multiple leads residing in the central venous system resulted in chronic superior vena cava syndrome. ICD = implantable cardioverter-defibrillator.

The patient is a 48-year-old man with a pertinent past medical history of HCM and atrial fibrillation/flutter. He initially had a left-sided dual-chamber implantable cardioverter-defibrillator (ICD) placed in 2002, which was later complicated by right ventricular (RV) lead fracture in 2017. Because of venous occlusion of the left subclavian venous system, the left-sided leads and system were abandoned and reimplanted on the right side at an outside facility. Unfortunately, this later led to right-sided subclavian stenosis and the development of symptomatic superior vena cava syndrome (Figure 1, Videos 1A and 1B). The patient's atrial fibrillation/flutter was initially managed with dofetilide, but in 2022, he had recurrence requiring an initial ablation (pulmonary vein isolation). Transthoracic echocardiogram in December 2022 showed a newly reduced LVEF of 15% with septal hypertrophy and global hypokinesis. He required 2 additional re-do ablations in 2023 for recurrent atypical atrial flutter before eventual arrhythmia quiescence that persists to the present. He was largely asymptomatic after re-do ablations despite reduced LVEF.

Starting in late 2023, the patient developed worsening heart failure symptoms despite maintaining sinus rhythm. He established care with an advanced heart failure team in early 2024 and was optimized on guideline-directed medical therapy. Despite medical optimization and rhythm control, the patient continued to be symptomatic with persistently reduced LVEF (20%). He was later hospitalized for decompensated heart failure, and a work-up for heart transplant was initiated. It was initially determined that the patient was a poor candidate for transplantation due to lack of superior venous conduits (Videos 1A and 1B). Around that time, the patient concurrently developed right-sided ICD lead fracture. To re-establish functional ICD therapy and in an attempt to facilitate transplant candidacy, a complex, multidisciplinary TLE and venous revascularization procedure was planned with an experienced, high-volume lead extractor.

Intravascular lithotripsy (Shockwave) was first applied in the distal superior vena cava from a femoral venous approach in an effort to break up calcium on the 4 indwelling leads. Bilateral lead extraction was performed from a superior approach using a combination of laser and mechanical cutting tools (Philips). Vascular access was unable to be re-established through the right subclavian system but was successfully maintained while extracting the left-sided leads. The vascular surgery team then performed repeated balloon angioplasty of the central venous system to re-establish patency (Video 2). At this juncture, several of the indwelling leads had fractured and the remnant parts were extracted in their entirety using a femoral snaring approach. A Boston Scientific subcutaneous ICD was then placed. Unfortunately, postprocedure transesophageal echocardiogram (TEE) revealed torrential TR and a new flail posterior tricuspid valve leaflet (Videos 3 and 4A). After initial stability, the patient quickly decompensated with cardiogenic shock from acute RV failure. He was transferred to the cardiac intensive care unit and was understandably deemed too high risk for open surgical correction of his acute primary TR.

The patient was presented emergently at multidisciplinary valve conference where the most appropriate intervention for the patient's acute TR was considered to be T-TEER using TriClip. Both traditional TEE and intracardiac echocardiography guidance as an adjuvant imaging modality were planned. Bilateral femoral venous access was obtained with a 24-F sheath and an 11-F sheath in the right and left common femoral veins, respectively. Four-dimensional intracardiac echocardiography was performed (Phillips VeriSight Pro). A 25-F steerable guide catheter was introduced into the right common femoral vein. The TriClip device was then introduced into the steerable guide catheter via a Clip Delivery system and positioned into the far posterior/septal canyon commissure. TEE and 3-dimensional multiplanar reconstruction were used to confirm precise clip placement and leaflet capture (Video 4B). Intracardiac echocardiography imaging was also used to further assess leaflet capture and device placement (Video 5). Once grasp was confirmed, the device was closed. Leaflet insertion, tissue bridge, and acceptable transvalvular gradients were then confirmed. Once these parameters were deemed satisfactory, the first TriClip was released. A second, planned, stabilizing TriClip was then placed in a similar fashion more centrally at the main pathology (Video 4C). TR severity measured after the placement of the second clip was +1, so the second clip was released (Videos 4D and 6).

## Why Beyond the Guidelines

To our knowledge, this case demonstrates the first use of T-TEER technology in the emergent management of acute torrential TR precipitating cardiogenic shock in a patient with end-stage HCM and severely reduced LVEF. There are limited data or guidelines for the nonsurgical management of acute TR, particularly when patients present in extremis with RV shock as a consequence of abrupt primary valve failure.

## Case Outcome and Follow-Up

After T-TEER, the patient was weaned off pressor support and discharged home days later. He was followed closely in the heart failure clinic, and his functional status improved. Given his stabilization in functional status and heart failure symptoms, he was no longer considered a candidate for heart transplantation.

## Discussion

To our knowledge, this is the first reported use of T-TEER for emergent correction of acute primary TR causing cardiogenic shock. Our patient developed primary TR as a complication of complex, laser-assisted TLE using relatively novel intravenous lithotripsy for obliteration of extensive fibrocalcific adhesions. Primary TR is a known, but rare, complication of TLE, occurring in approximately 0.1% of cases.^4^ It is generally well tolerated and considered a minor complication. However, in patients with pre-existing right and/or left ventricular dysfunction, acute reduction in left ventricular preload can be life threatening. Because primary unstable TR is rare, there are no guidelines to inform management.

Virtually all patients included in the pivotal T-TEER trials had functional or degenerative TR, and none had severe left ventricular dysfunction or shock.^2^^,^^3^ However, T-TEER may be well suited for the emergent treatment of acute primary tricuspid valve failure. Acute primary valve failure is associated with smaller valve leaflet bridge distances than chronic functional disease, which makes leaflet capture easier than in cases with functional disease. In addition, in contrast to valve replacement, T-TEER does not completely eliminate TR. Recent data demonstrate the Evoque (Edwards) valve's efficacy for severe TR, and valve replacement likely offers a more durable solution than repair.^5^ However, total elimination of TR can result in acute afterload mismatch, which may precipitate new or worsening RV failure. T-TEER, therefore, may be uniquely beneficial for treating primary TR in patients with tenuous hemodynamics because it allows the tricuspid valve to retain its “pop-off valve” properties.

Currently, there are 2 major T-TEER systems: TriClip (Abbott) and PASCAL (Edwards). PASCAL is not yet commercially available in the United States. A recent meta-analysis showed TriClip's superiority in reducing vena contracta width compared with PASCAL and MitraClip (Abbott), suggesting that the system may be more effective in repairing severe or torrential TR.^6^ The same study found that PASCAL involves longer fluoroscopy time than TriClip, which is suboptimal for patients who are unstable and need rapid correction. TriClip, therefore, may be preferable to PASCAL for emergent management of acute, torrential TR.

## Conclusions

T-TEER with TriClip could be used in patients with RV shock from acute torrential TR and severe left ventricular dysfunction.

## Funding Support and Author Disclosures

Open access publication fees for this article were provided by Sentara Health, Virginia Beach, VA. Dr Yehya has received speaking honoraria and consulting fees from Bayer, Merck, Novo Nordisk, AstraZeneca, Bridgebio, and scPharmaceuticals. Dr Kiehl has received consulting, advisory board, and proctoring income from Phillips. Dr Summers has received speaking, consulting, and proctoring income from Medtronic, Abbott Pharmaceuticals, Abiomed, and Shockwave. All other authors have reported that they have no relationships relevant to the contents of this paper to disclose.

**Take-Home Messages**

• Transcatheter tricuspid edge-to-edge repair is a reasonable modality to treat acute, severe tricuspid regurgitation.
• Transcatheter tricuspid edge-to-edge repair may be feasible in stable patients with severe left ventricular dysfunction and ejection fractions lower than those represented in the pivotal trials.

## Appendix

Video 1 Fluoroscopic Venogram of Superior Central Venous System (A) Fluoroscopic venogram showing stenosis of the right subclavian and innominate systems and superior vena cava (SVC). (B) Fluoroscopic venogram showing stenosis of the left subclavian and innominate systems and SVC.

Video 2 Central Venography Following Balloon Angioplasty Remnants of right atrial leads and left-sided right ventricular lead are shown.

Video 3 TEE Transgastric View of Acute Torrential TR With Flail Posterior Tricuspid Leaflet TEE = transesophageal echocardiogram; TR = tricuspid regurgitation.

Video 4 Intraprocedural Echocardiography During T-TEER (A) Torrential tricuspid regurgitation on intraprocedural TEE before TriClip deployment. (B) Initial TriClip position, leaflet capture, and bridging confirmed via TEE and 3-dimensional multiplanar reconstruction. (C) Position and capture confirmation of the second TriClip device. (D) Final regurgitation jet after placement of 2 sequential TriClips (+1). TEE = transesophageal echocardiogram.

Video 5 Intracardiac Echocardiography Visualization of Leaflet Capture and Device Placement for the First TriClip Device

Video 6 TEE Transgastric View Showing the Final Position of 2 TriClip Devices TEE = transesophageal echocardiogram.
